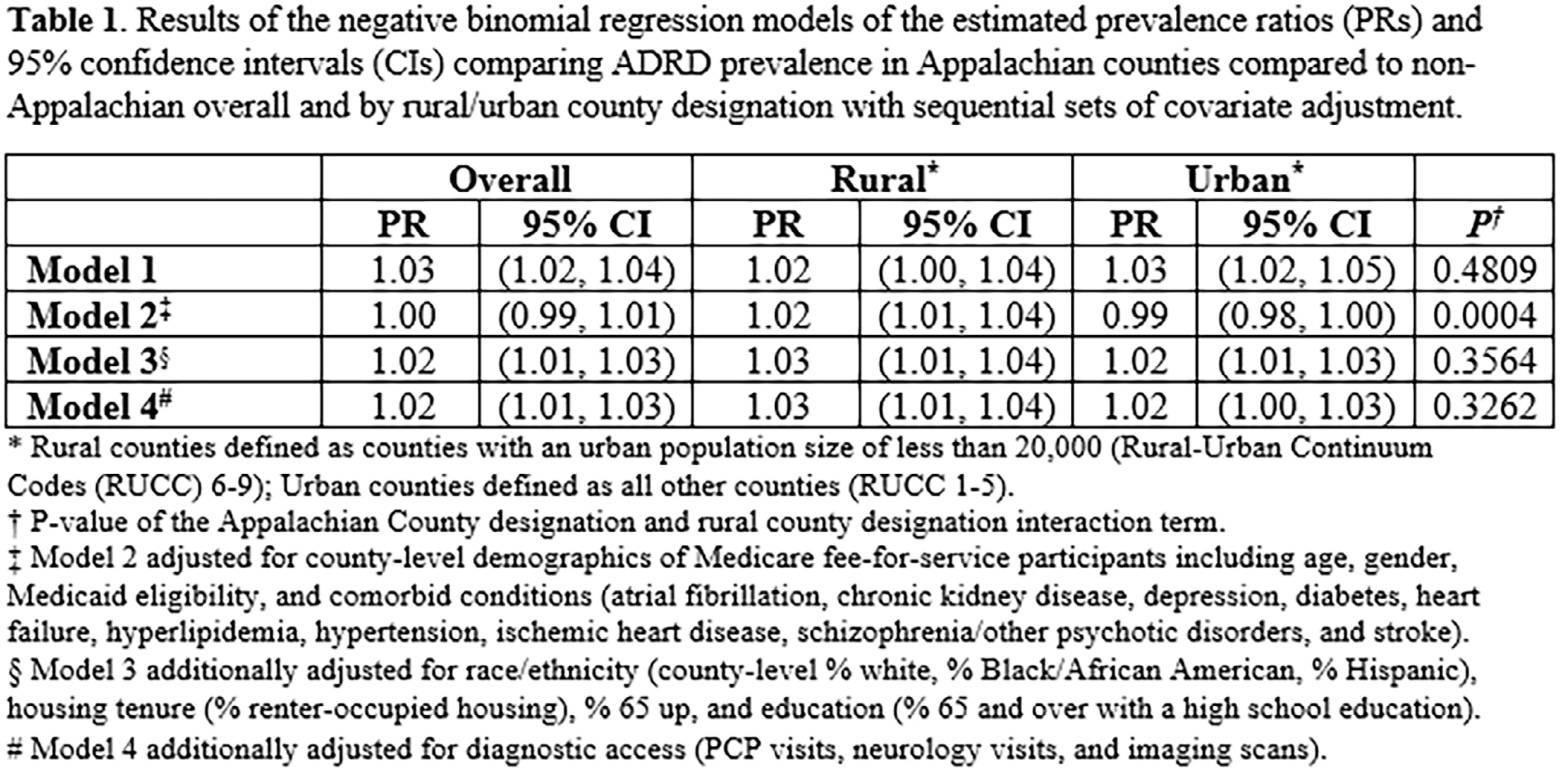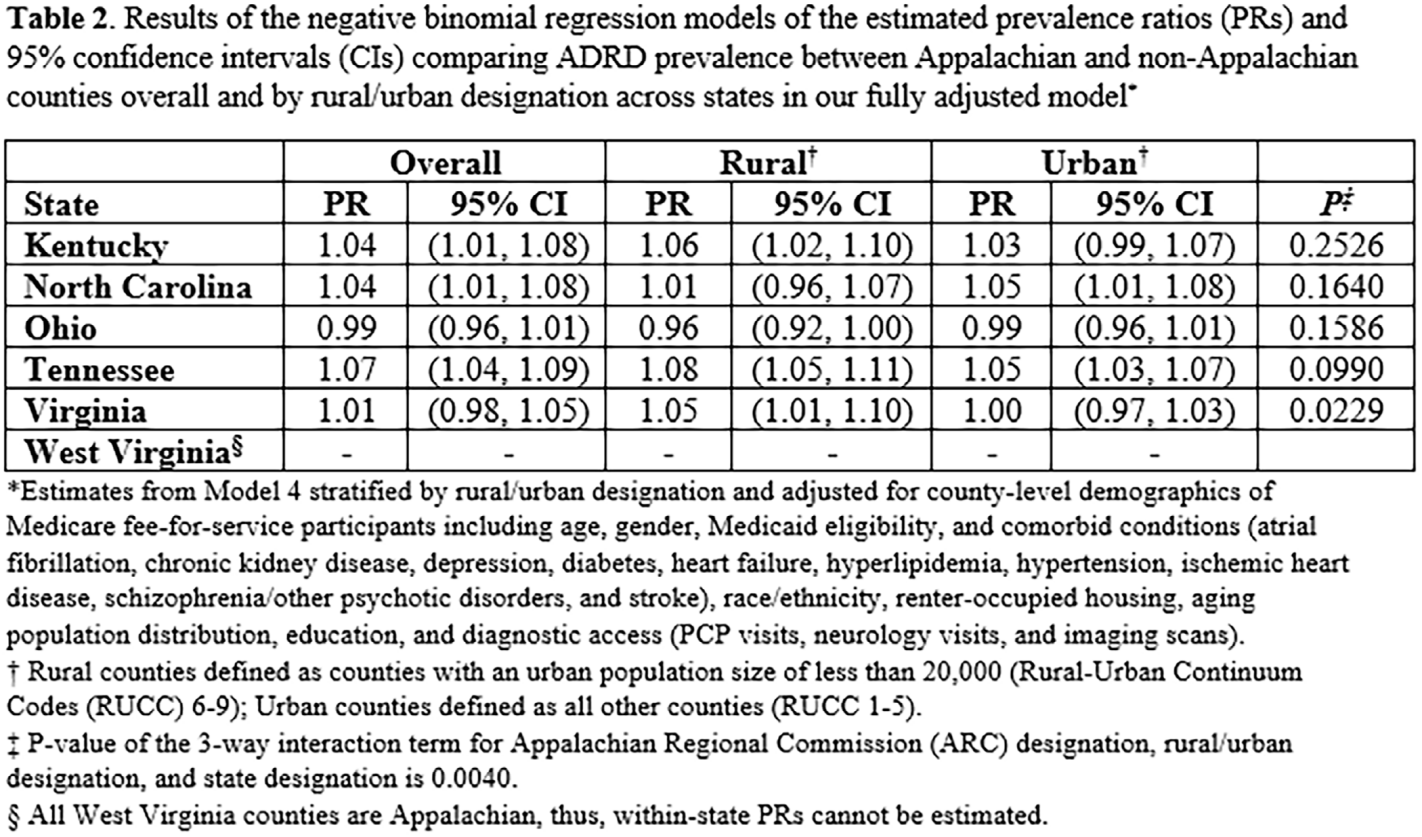# Alzheimer’s Disease and related dementias prevalence higher in Appalachian counties within Central Appalachia

**DOI:** 10.1002/alz.087720

**Published:** 2025-01-09

**Authors:** Jeffrey Wing, Jenna Rajczyk, James Burke

**Affiliations:** ^1^ Ohio State University, Columbus, OH USA

## Abstract

**Background:**

Alzheimer’s disease and related dementias (ADRD) prevalence varies geographically in the US. The Appalachian region has lower educational attainment and health care access barriers compared to non‐Appalachian regions. The objective of this proposal is to assess whether the geographic variation of ADRD in Central Appalachia is explained by county‐level sociodemographic factors or access to care.

**Methods:**

Centers for Medicare and Medicaid Services Public Use Files from 2015‐2018 were used to estimate county‐level ADRD prevalence among all fee‐for‐service beneficiaries in Central Appalachia (Kentucky, North Carolina, Ohio, Tennessee, Virginia, and West Virginia). Negative binomial regression was used to estimate prevalence overall, by Appalachian Regional Commission’s Appalachian/non‐Appalachian designation, and by rural/urban (Rural‐Urban Continuum Codes) classification. Models were then adjusted for: (1) county‐level demographics including age, gender, Medicaid eligibility, and comorbidities (atrial fibrillation, chronic kidney disease, depression, diabetes, heart failure, hyperlipidemia, hypertension, ischemic heart disease, schizophrenia/other psychotic disorders, and stroke); (2) sociodemographics (race/ethnic distribution, education, aging population distribution, and renter‐occupied housing); and (3) diagnostic access (PCP visits, neurology visits, and imaging scans).

**Results:**

A total of 592 counties comprise the Central Appalachian region where 43% (n = 255) are Appalachian and 45% (n = 267) are rural counties. The prevalence of ADRD did not vary over the time period but was higher for Appalachian counties both overall (PR: 1.03; 95% CI: 1.02, 1.04) and after adjustment (PR: 1.02; 95% CI: 1.00, 1.03) compared to non‐Appalachian counties. This difference was similar among rural and urban counties (*p* = 0.326). These differences did vary across the states in the region *(p* = 0.0040). In North Carolina and Tennessee, Appalachian counties had higher ADRD prevalence, while in Ohio and Tennessee, rural counties have lower ADRD prevalence.

**Conclusion:**

There is a consistent difference between Appalachian and non‐Appalachian counties, with Appalachian counties having relatively higher ADRD prevalence (in the range of 2.0‐3.0% times higher) than non‐Appalachian counties. These differences persisted even after considering age, education, other sociodemographic factors and access to care measures.